# The Comparison of In Vitro Photosensitizing Efficacy of Curcumin-Loaded Liposomes Following Photodynamic Therapy on Melanoma MUG-Mel2, Squamous Cell Carcinoma SCC-25, and Normal Keratinocyte HaCaT Cells

**DOI:** 10.3390/ph14040374

**Published:** 2021-04-17

**Authors:** Marta Woźniak, Martyna Nowak, Anastasiia Lazebna, Kamil Więcek, Izabella Jabłońska, Krzysztof Szpadel, Aleksandra Grzeszczak, Jerzy Gubernator, Piotr Ziółkowski

**Affiliations:** 1 Department of Pathology, Wroclaw Medical University, 50-367 Wroclaw, Poland; martyna.nowak96@o2.pl (M.N.); jabizabella@gmail.com (I.J.); szpadelk@gmail.com (K.S.); piotr.ziolkowski@umed.wroc.pl (P.Z.); 2 Department of Biotechnology, University of Wroclaw, 50-383 Wroclaw, Poland; stoneqer@gmail.com (A.L.); kamil.wiecek24@gmail.com (K.W.); 3 Department of Lipids and Liposomes, Faculty of Biotechnology, University of Wroclaw, 50-383 Wroclaw, Poland; aleksandra.grzeszczak@uwr.edu.pl

**Keywords:** curcumin, natural photosensitizer, photodynamic therapy, skin cancer treatment, squamous cell carcinoma, melanoma, normal keratinocytes, liposomes

## Abstract

The research focused on the investigation of curcumin encapsulated in hydrogenated soy phosphatidylcholine liposomes and its increased photoactive properties in photodynamic therapy (PDT). The goal of this study was two-fold: to emphasize the role of a natural photoactive plant-based derivative in the liposomal formulation as an easily bioavailable, alternative photosensitizer (PS) for the use in PDT of skin malignancies. Furthermore, the goal includes to prove the decreased cytotoxicity of phototoxic agents loaded in liposomes toward normal skin cells. Research was conducted on melanoma (MugMel2), squamous cell carcinoma (SCC-25), and normal human keratinocytes (HaCaT) cell lines. The assessment of viability with MTT (3-(4,5-dimethylthiazolyl-2)-2,5-diphenyltetrazolium bromide) evaluated cell death after exposure to blue light irradiation after 4 h of pre-incubation with free and encapsulated curcumin. Additionally, the wound healing assay, flow cytometry, and immunocytochemistry to detect apoptosis were performed. The malignant cells revealed increased phototoxicity after the therapy in comparison to normal cells. Moreover, liposome curcumin-based photodynamic therapy showed an increased ratio of apoptotic and necrotic cells. The study also demonstrated that nanocurcumin significantly decreased malignant cell motility following PDT treatment. Acquired results suggest that liposomal formulation of a poor soluble natural compound may improve photosensitizing properties of curcumin-mediated PDT treatment in skin cancers and reduce toxicity in normal keratinocytes.

## 1. Introduction

Skin cancers are among the most widespread types of neoplasm, affecting people from less pigmented, Caucasian populations, usually more than 50 years of age [1]. Those malignancies are divided into two main subgroups composed of more lethal melanoma and more prevalent non-melanoma skin cancers (NMSC). Currently, non-melanoma skin cancers are generally represented by tumours from transformed keratinocytes: cutaneous squamous cell carcinoma (cSCC) and basal cell carcinoma (BCC). There are also other skin-related neoplasms, including Kaposi’s sarcoma, Merkel cell and adnexal carcinoma, cutaneous lymphoma, or dermatofibrosarcoma protuberans. Nevertheless, those conditions are not as common as NMSCs [2,3]. Like most tumours, skin cancer treatment involves widely used methods, such as surgical intervention, radiotherapy, chemotherapy, and immunotherapy. However, despite the effectiveness of the former, it cannot be applied in all cases due to adverse malignancy’s localization and potential patient’s co-morbidities [2]. Moreover, radiotherapy and chemotherapy can not only be toxic and lead to side effects but also might be ineffective in cells with developed resistance [4], whereas immunotherapy currently seems too complex for general use [5]. Therefore, further development of more efficient therapeutic strategies is still required.

One of the novel approaches that could potentially overcome those obstacles is the usage of photodynamic therapy (PDT). The main principle of this method is to apply and accumulate the chosen substance with photosensitive properties (called further photosensitizer (PS)) within tumour tissue. Later, after local irradiation with a specific wavelength laser, excited PS can transform surrounding molecules into highly active reactive oxygen forms (ROS). Depending on the mechanisms, excited PS may transfer electrons on organic compounds (via type I reaction), creating radicals such as hydrogen peroxide, or transmits its energy on molecular oxygen by developing a singlet oxygen (^1^O_2_) (via type II reaction) [6]. Accumulated ROS can damage plenty of biomolecules, including lipids, proteins, DNA, and carbohydrates. However, due to the limited diffusing capabilities of radicals, their damaging properties rely on PS. Whether PS exhibits a higher affinity to concentrate closely to mitochondria’s membrane or enzymes, radicals’ activities may have a different impact on cells. ROS and singlet oxygen from an activated photosensitizer can induce cell death, mainly by damaging lipids of plasma and organelles membranes, triggering caspases cascade, and inactivating anti-apoptotic proteins. Depending on the efficiency of PS, photokilling can occur by rough conditions of necrosis or (preferably) in milder cases apoptosis or/and autophagy [7,8,9].

Photodynamic therapy is gaining growing interest due to its low invasiveness, high selectivity, and comparable lower costs to other treatments. Nonetheless, presently, PDT is not applicable in the treatment of metastatic cells [10]. The most common PS evoke a low therapeutic effect against highly pigmented melanoma cells [5], and the method itself can be burdened with pain, especially in combination with commonly used 5-aminolevulinic acid (5-ALA) [4]. For all those reasons, PDT enhancement is currently extensively investigated, especially with novel, high-efficient, and less toxic photosensitizers.

Curcumin is a natural polyphenol extracted from turmeric (*Curcuma longa*), with well-documented anti-tumour, anti-inflammatory, and photoactive properties [11,12,13]. This golden polyphenol has already been used for its anti-inflammatory effects, as a treatment in various dermatological conditions [14].

Due to its exceptional attributes, this plant-derived substance could potentially play a dualistic role in PDT functioning simultaneously as PS and a direct therapeutic molecule. Experiments conducted on animal models and in vitro suggest that curcumin can downregulate various molecular responses in boosting up inflammatory and pro-survival pathways, such as those related to transcription factors like Nf-κB or AP-1 [15,16]. Thus, curcumin could potentially not only increase the chances of apoptosis in defective cells but also stimulate the production of cell killing radicals, making it a promising compound to use in PDT therapies.

Among all the previously mentioned benefits, the extremely poor water solubility and low bioavailability of this natural plant derivative limit its clinical use in cancer treatment. Moreover, basic skin properties made it an excellent barrier decreasing percutaneous penetration of curcumin. For this reason, the development of stable formulations of drug carriers that improve skin penetration and therapeutic effectiveness with reduced side effects is an essential challenge for many researchers [17]. Nowadays, various nanocarriers, which could greatly enhance the bioavailability of drugs, are under intensive development and some of them had already been functionalized for active targeting of skin cancers, including those based on gels or liposomes that are modified with aptamers [18,19].

According to several studies, nano-formulations of photosensitizers improve the pharmacokinetic effects and therapeutic advantage of free compounds [20,21,22]. Besides that, lipid formulations are proposed as an alternative strategy to potentiate the effect of PDT against resistant melanoma cells [23]. Liposomes are a versatile drug delivery system. They are not toxic and (when pegylated) exhibit longer circulation time among all drug carriers. Liposomes can encapsulate hydrophilic, hydrophobic, and amphiphilic molecules. Liposomes have many advantages, such as controlled release properties, cell affinity, tissue compatibility, reducing drug toxicity, and improving drug stability. As most drug carriers, liposomes can accumulate in inflammatory tissues by using the enhanced permeability and retention (EPR) effect. This accumulation can be further increased by decreasing particle size as well as pretreatment by some drugs and substances [24,25,26,27].

In general, at least in the animal model, an essential increase of drug concentration is observed in the tumour tissue when liposomal drugs are applied. In the case of curcumin, which is a hydrophobic, the use of liposomes may diminish issues with its low solubility and bioavailability, enhancing pharmacokinetics and accumulation in cancer tissues [16,20,21].

In this study, a relatively novel curcumin formulation has been used, in which curcumin is encapsulated in liposomes composed from hydrogenated soy phosphatidylcholine (HSPC), which exhibited high stability, due to a relatively rigid liposomes’ bilayer and, therefore, low curcumin diffusion properties. This formulation proved its superiority in comparison to a free substance on pancreatic cancer cell lines and can be regarded as an essential improvement of the traditional route of curcumin supply [28,29,30,31].

**Figure 1:**
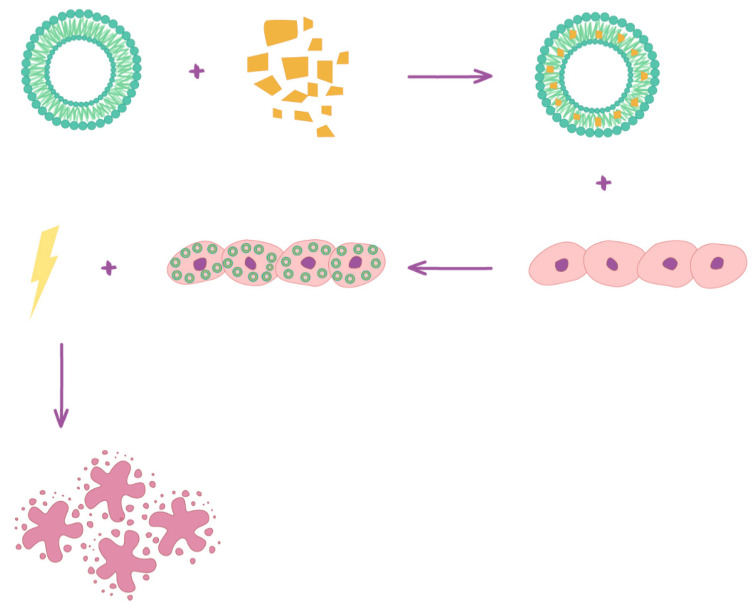
Scheme of encapsulated curcumin in liposomes and mechanism of photodynamic therapy.

Herein, a comparison of the phototoxic and anti-cancerous effects of curcumin and its stable HSPC liposomal formulation on skin cancer cell lines was conducted, including SCC-25 representing cutaneous squamous cell carcinoma, MUG-Mel2 representing a melanoma cell line, and normal human keratinocytes HaCaT representing control normal skin cells (Figure 1). To evaluate the effects of encapsulated curcumin as a photosensitizer in PDT on different skin cell lines, MTT (3-(4,5-dimethylthiazolyl-2)-2,5-diphenyltetrazolium bromide) dark cytotoxicity, phototoxicity assay, immunocytochemical staining against markers of apoptosis, Bcl-2, and Bax, measuring apoptosis with flow cytometry and a wound-healing assay, were performed.

## 2. Results

### 2.1. The Effect of Curcumin and Liposomal Curcumin Based PDT on MUG-Mel2, SCC-25, and HaCaT Cells Viability Measured by an MTT Assay

**Figure 2:**
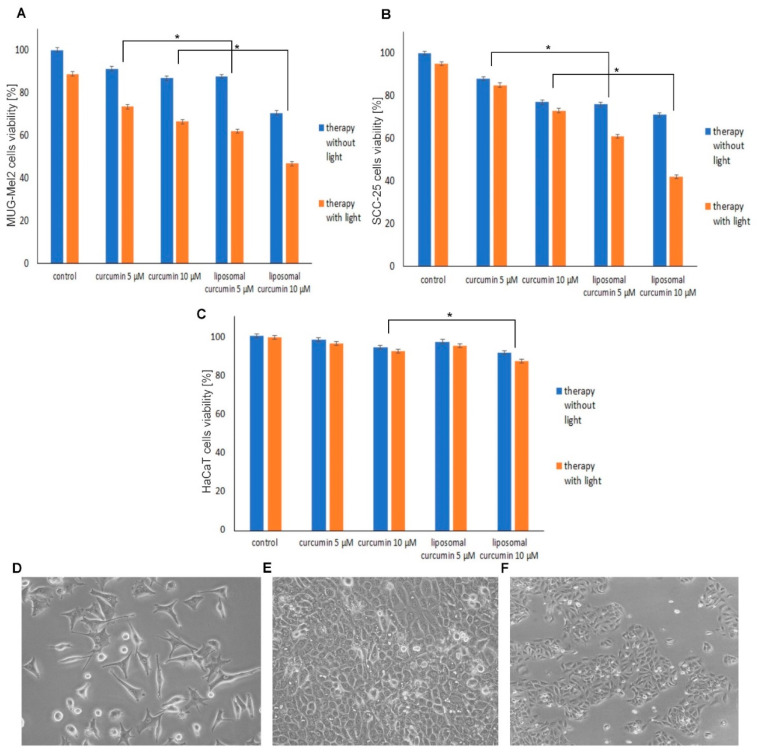
Results of cell viability after 4 h of incubation with 5, 10 µM curcumin and liposomal curcumin with and without the irradiation (2.5 J/cm^2^) evaluated by the MTT (3-(4,5-dimethylthiazolyl-2)-2,5-diphenyltetrazolium bromide) assay. (**A**) Viability-dependent bars for MUG-Mel2 cells after incubation with curcumin and liposomal curcumin without/with light. (**B**) Viability-dependent bars for SCC-25 cells after incubation with curcumin and liposomal curcumin without/with the light. (**C**) Viability-dependent bars for HaCaT cells after incubation with curcumin and liposomal curcumin without/with light. Encapsulated curcumin is significantly more cytotoxic than free curcumin in cancer cells. (**D–F**) Representative images of MUG-Mel-2, SCC-25, HaCaT cells morphology detected by phase-contrast microscopy. Results represent the mean from three different experiments. * *p* < 0.05.

The effect of curcumin and liposome-curcumin-based PDT was performed on skin cancer cell MUG-Mel2 (melanoma cells), SCC-25 (squamous cell carcinoma), and normal keratinocyte cells HaCaT. Effectiveness of free and encapsulated curcumin was compared in doses of 5 and 10 μM after blue light low irradiation (2.5 J/cm^2^). Results indicated that liposome curcumin-mediated PDT caused a significantly higher reduction of viability in both cancer cell lines than a free natural compound. Curcumin mediated-PDT in 10 μM concentration caused decreased viability in SCC-25 (34%) and MUG-Mel2 (27%). Liposomes with curcumin mediated-PDT inhibited cancer cells’ growth more than a free compound after irradiation reaching IC50. Liposomal-curcumin-PDT exhibit cytotoxicity of 53% in MUG-Mel2 and 58% in SCC-25 at the same dose—10 μM and low irradiation dose (2.5 J/cm^2^) while the viability of HaCaT was decreased only by 11%. Interestingly, HaCaT cells maintained viability of around 90% after different treatments. Liposomal curcumin in the concentration of 10 μM was chosen in all subsequent biological studies (Figure 2).

### 2.2. The Effect of Liposomal Curcumin Based PDT on MUG-Mel2, SCC-25, and HaCaT Cells in the Wound-Healing Process

**Figure 3:**
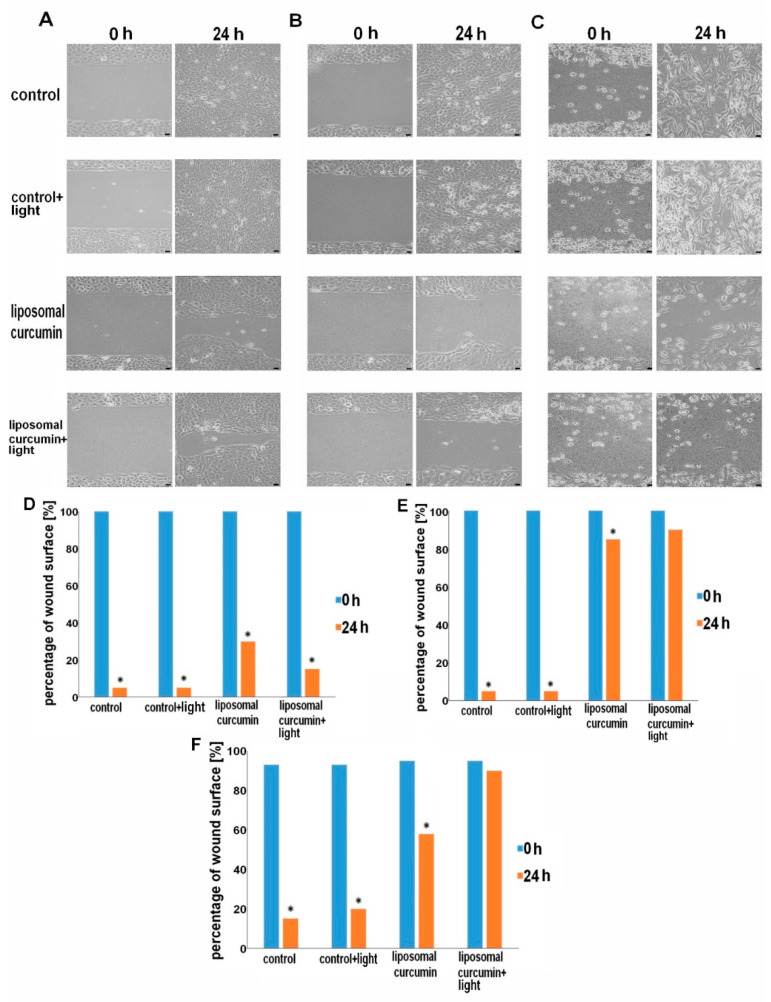
Wound-healing assay in time point 0 h and 24 h of (**A**) HaCaT, (**B**) SCC-25, and (**C**) MUG-Mel2 cell line. Representative images show that, after 24 h, the scrap in control cells is minimal compared to cancer cells treated with liposomal curcumin in dose 10 µM with blue light (2.5 J/cm^2^). In treated cells, in the HaCaT control cell line, the scrap is smaller than in the other two cancer cells (**D**–**F**). (**D**) Quantification of cell migration for HaCaT, SCC-25, and MUG-Mel2 cells. Results are presented as the percentage of the wound surface. The initial wound area is expressed as 100% at 0 h. Results represent the mean from three different experiments. Scale bar = 50 µm. * *p* < 0.05.

To check whether liposomal curcumin-based PDT decreases HaCaT, SCC-25, and MUG-Mel2 cells’ motility, the wound healing test was performed. The assay shows the migration of cells by evaluating a primaeval scratch’s closure in a 24 h observation. The results show that PDT with liposomal curcumin caused the strongest effect of migration properties in MUG-Mel2 cancer cells. After 24 h from the treatment (liposomal curcumin and the light), there was no migration observed. In SCC-25 cells, the wound was minimally closed, whereas, in normal HaCaT cells, the wound closed almost entirely within 24 h of incubation after therapy. The results are presented in Figure 3.

### 2.3. The Effect of Liposomal Curcumin-Based PDT on MUG-Mel2, SCC-25, and HaCaT Cells on Bax and Bcl-2 Expression

**Figure 4:**
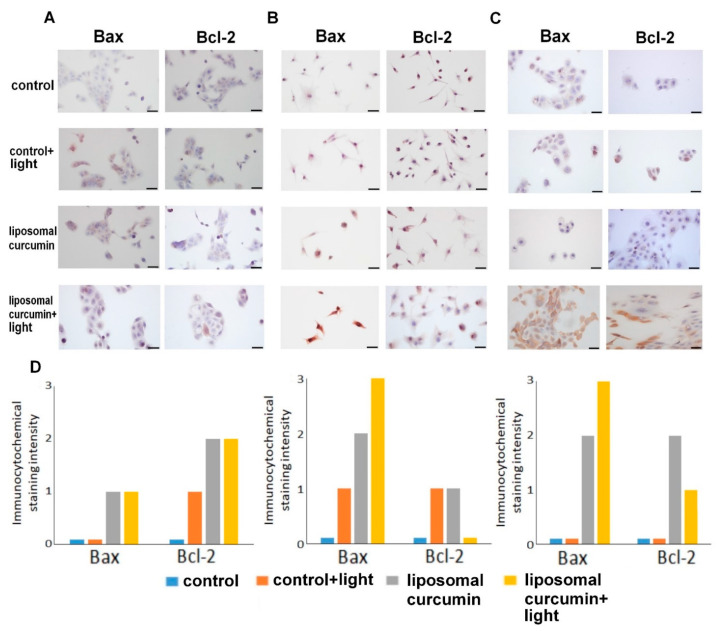
Presenting immunocytochemical staining of the chosen apoptosis-related proteins Bax and Bcl-2 in HaCaT (**A**), MUG-Mel2 (**B**), and SCC-25 (**C**) cells lines in four conditions: control, control with blue light only, liposomal curcumin in dose 10 µM, and liposomal curcumin in dose 10 µM with a low dose of blue light (2.5 J/cm^2^). (**D**) Results of immunocytochemical analysis of HaCaT, MUG-Mel2, and the SCC-25 cell line and the chosen apoptosis-related protein expression calculated by the immunoreactivity score. Abbreviations: 0-no staining, 1-weak staining, 2-moderate staining, and 3-strong staining. Scale bar = 50 μm.

Immunocytochemical staining allows examining whether the proposed therapy with liposomal curcumin and irradiation has a cytotoxic effect on cancer cells. To assess whether the treatment causes apoptosis in cancer cells, apoptosis-related proteins bax and bcl-2 were used for the immunocytochemical analysis and then evaluation of immunoreactivity was performed. An increase in the expression of bax and decreased expression of bcl-2 in cancer cells, MUG-Mel2 and SCC-25, was observed (Figure 4). In both cancer cell lines, pro-apoptotic bax protein showed strong expression after treatment of cells with liposomal curcumin and irradiation. The expression of bcl-2 was weak or moderate. Nonetheless, HaCat cells did not significantly change the expression of the previously described proteins after irradiation only, liposomal curcumin only, and PDT treatment.

### 2.4. The Impact of Liposomal Curcumin on Cells Lines’ Apoptosis

**Figure 5:**
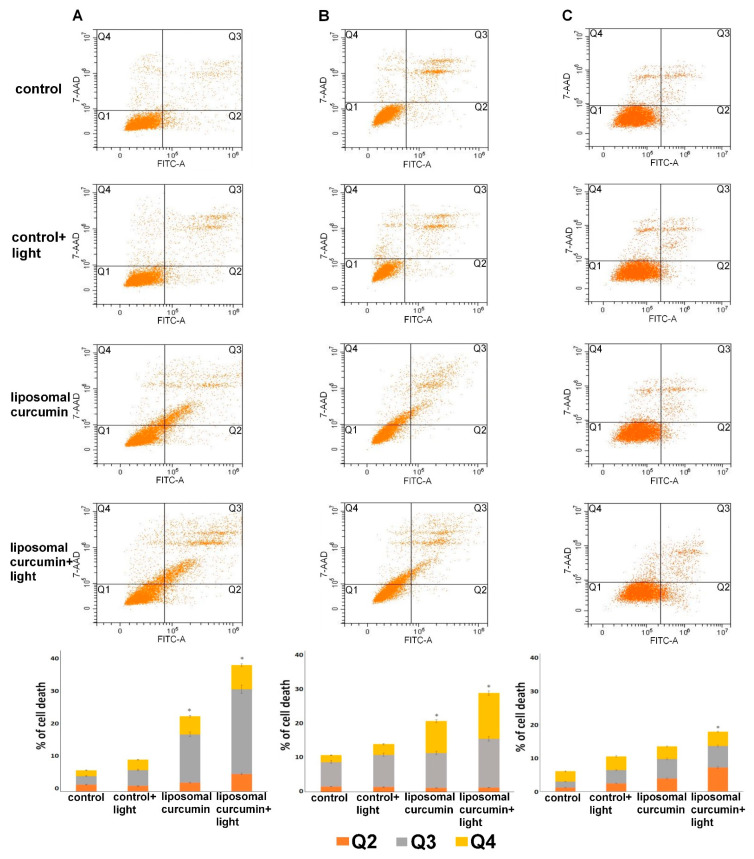
Effect of photodynamic therapy (PDT) with liposomal curcumin in dose 10 µM with blue light (2.5 J/cm2) on SCC-25 (**A**), MUG-Mel2 (**B**), and HaCaT (**C**) cells. Dot plots present alive-Q1, early apoptotic-Q2, late apoptotic-Q3, and dead-Q4 cells. After therapy, the cells were stained using Annexin-FICT /7-AAD Kit and were measured by flow cytometry. Bars represents the quantitative percentage of total apoptotic cells (early + late apoptosis) and necrotic cells in SCC-25, MUG-Mel2, and HaCaT cells. Results represent the mean from three different experiments. * *p* < 0.05.

Flow cytometry analysis was applied to evaluate cell death caused by liposomal curcumin in SCC-25, MUG-Mel2, and HaCaT cells (Figure 5). As shown in Figure 4A and Figure 4B after 24 h of treatment, early and late apoptosis and necrosis in SCC-25 and MUG-Mel2 cells were observed. The combination of liposomal curcumin and PDT increased apoptosis to 40% and 30% in SCC-25 and MUG-Mel2 cells, respectively. Interestingly, after 24 h from irradiation, in SCC-25, cell death is mainly caused by early and late apoptosis, whereas, in MUG-Mel2, cell death is caused by late apoptosis and necrosis. In control cells, HaCaT, a slight increase in the apoptosis ratio in cells after treatment (10%) was observed.

## 3. Discussion

In the past, various approaches were undertaken in order to increase the efficacy of photodynamic therapy [32,33]. The studies included the application of chemically functionalized PS [34,35,36] as well as liposomal derivatives of photosensitizers for both in vitro and in vivo studies. Several studies showed an advantage of the latter modality over the routine way of photosensitizer delivery to targeted cells [37,38]. Curcumin, which revealed promising effects in PDT, can act as a direct photosensitizer exhibiting cytotoxic properties in various types of tumours, including skin cancers [12,39,40].

Although curcumin can be applied in a pure form and then sensitized with light at the proper wavelength, its liposomal formulation was proposed as the more effective strategy in killing the malignant cells [41,42]. Free curcumin is characterized by low water solubility and poor bioavailability. It is rapidly metabolized or degraded in the cell culture media or after oral administration. In contrast, nano-capsules in which the compound is confined into phospholipid bilayers dismiss the significant drawbacks and promote increased absorption of curcumin into the cells [43].

In the present study, the effectiveness of curcumin loaded in PEGylated, cholesterol-free formulation based upon hydrogenated soya PC liposomes has been investigated on three skin cell lines: melanoma MUG-Mel2, squamous cell carcinoma SCC-25, and immortalized keratinocytes HaCaT cells. In previous studies, the previously described formulation of liposomes was evaluated on the pancreatic cell line and in human plasma [31]. The results indicated that this formulation presented the best parameters of the hydrophobic drug incorporation by improved bioavailability, increased stability, and cytotoxicity. In this article, the MTT results revealed statistically significant phototoxicity of this liposomal formulation of 10 µM curcumin compared to the free substance before and after photodynamic therapy. In the case of a free substance, it interacts with the cellular outer membrane, while liposomes are quickly internalized and enter the cell through the endosomal route, which increases its bioavailability and, thus, results in more potent cytotoxic effects [43,44]. Acquired results are in accordance with the observations and conclusions of Vetha et al. and Ambreen et al. on different cancer cell lines [41,45]. Although the effect was evident for both malignant cell lines, normal HaCaT keratinocytes were slightly resistant to the therapy. These spontaneously immortalized human keratinocytes from adult skin have been used as a model cell line to study normal keratinocyte functions in different studies [46]. Additionally, HaCaT cells maintained in a culture medium without the calcium display normal morphogenesis and expression of the cellular membrane markers as keratinocytes isolated from adult skin [47]. Based on gleaned, different experimental results, conclusions emerge that immortalized HaCaT keratinocytes are less susceptible to photosensitization with curcumin than MUG-Mel2 and SCC-25 malignant cells in terms of phototoxicity. These observations are following the results of Popovic et al. [48]. The authors found that 3 μM hypercin-mediated-PDT is completely refractory to keratinocytes. Moreover, they indicated a different response toward a natural plant derivative compound-PDT in each skin cell type. On the other hand, Szlasa et al. [12] presented the increased cytotoxic impact of the free curcumin-mediated photodynamic therapy on the keratinocytes. However, according to the cell line and light dose used in their studies described in the methods paragraph, the authors used normal human epidermal keratinocytes (HEK) and 6 J/cm^2^ to irradiate cells in their experiments. These differences in the cells’ response to curcumin irradiated with blue light may be considered due to the distinct vulnerability of cell lines to the cell-stress induction and different PDT protocols.

The present study results showed that liposomal formulation of a compound considered a potent photosensitizer can also enhance the effectiveness of liposomal curcumin-mediated-PDT by increasing the apoptosis ratio validated by flow cytometry and the production of pro-apoptotic factors, e.g., Bax protein. At the same time, the proposed therapy decreases the production of anti-apoptotic proteins, which is, in this case, Bcl-2. The significantly increased strong Bax expression was observed in both cancer cell lines, whereas, in HaCaT cells, Bax expression was lower in the sample treated with liposomal curcumin irradiated with the light. A flow cytometry assay confirmed this effect. Cells were stained with Annexin V-FITC and propidium iodide to detect early and late apoptosis and dead cells after treatment. It has been noticed that the late apoptosis in SCC-25 and MUG-Mel2 cells was increased after 24 h from the proposed therapy. Interestingly, SCC-25 cells revealed apoptosis as a leading cause of cell death, while MUG-Mel2 cells showed both types of cell death as a possible mechanism. The above finding remains in concordance with the results of other authors and shows that, in hydrophobic photosensitizers, an increase of photodynamic efficacy could be achieved by trapping them in liposomes [41,45].

The presented observations also point toward a possible mechanism of action of curcumin in PDT via an apoptotic pathway. Cells in all three examined groups showed necrosis, which is routinely observed after the PDT [7,49].

As a result of the different proliferative and migration capabilities of examined skin cell lines, a designed treatment on migration potency by a wound healing assay has been evaluated. A further examination confirms a decreased motility of melanoma and squamous cell carcinoma cell lines compared to normal keratinocytes after liposomal curcumin only and liposomal curcumin following irradiation, which is consistent with Szlasa et al. examination of the wound [12]. Normal cells nearly filled the wound (15% remaining) by 24 h, whereas the wound in malignant cells remained unfilled after 24 h. According to Ambreen et al., it is evident that liposomal curcumin-PDT reduces cancer cell migration and contributes to malignant cell metastasis inhibition.

Conducted investigations indicate the promising role of curcumin encapsulated in hydrogenated soy phosphatidylcholine liposomes in enhancing the photokilling effect on melanoma and squamous skin cancer cells following blue light PDT. Additionally, a minimal phototoxic reaction was observed in normal, human, immortalized keratinocytes with the same curcumin dose after irradiation. In conclusion, further experiments on the specific, cellular functional differences between the skin cells and in vivo testing will help confirm the effectiveness of nanocurcumin as a photosensitizer in PDT.

## 4. Materials and Methods

### 4.1. Cell Culture

Melanoma MUG-Mel2 (DSMZ, Germany) cells were cultured in RPMI 1640 cell culture medium, SCC-25-tongue squamous carcinoma (DSMZ, Braunschweig, Germany) cells in DMEM-F12, and HaCaT human epidermal keratinocytes (CLS, Eppelheim, Germany) were cultured in DMEM (Dulbecco’s Modified Eagle Medium) without calcium to maintain normal morphogenesis and expression of the cellular membrane markers. To prepare a full cell culture media, 10% FBS, 1% glutamine, and 1% antibiotics were added to the bottle. Culture reagents were bought from Gibco (Thermo Fisher Scientific Inc., Waltham, MA, USA). Cells were maintained at 37 °C and 5% CO_2_ in a humidified atmosphere. For experiments, cells from the 3rd to the 10th passages were used.

### 4.2. Preparation of Curcumin-Loaded Liposomes and Curcumin in DMSO

Curcumin-loaded liposomes of the composition HSPC/DSPE-PEG2000 9.5:0.5 mol/mol were formulated using the extrusion technique. Hydrogenated soy phosphatidylcholine (Phospholipon 90H, HSPC), 1,2-distearol-sn-glycero-phosphoethanolamin-N-(poly[ethylene glycol]2000) (DSPE-PEG2000) were purchased from Lipoid GmbH (Ludwigshafen, Germany). In brief, lipids and curcumin were dissolved in chloroform or methanol to obtain stock solutions at 10 and 5 mg/mL, respectively. Curcumin (2 mg) was mixed together with 40 mg of lipid in a borosilicate glass tube. Solvents were removed from the sample via evaporation under a stream of nitrogen gas and the resultant lipid film was dissolved in a mixture of cyclohexane and methanol (99:1, *v*/*v*). The sample was frozen in liquid nitrogen and freeze-dried for 8 h at a low pressure using a Savant Modulyo apparatus (Thermo Fisher Scientific, Waltham, CA, USA). The lipid film was hydrated by the addition of 1.5 mL of 150 mM NaCl at 64 °C, in a water bath, with gentle mixing. The liposomal suspension was finally sonicated in a water bath sonicator for 8 min at 64 °C. The newly-formed multilamellar vesicles (MLVs) were extruded 10 times through Nucleopore polycarbonate filters (Whatman, Maidstone, UK) with pore sizes of 400 and 100 nm, respectively, using a Thermobarrel Extruder (10 mL Lipex extruder, Northern Lipids, Canada) to obtain large uni-lamellar vesicles (LUVs). The extruder was maintained at 64 °C throughout the liposome extrusion procedure.

The curcumin: (1E, 6E)-1,7-bis-(4-hydroxy-3-methoxyphenyl)-1,6- heptadiene-3,5-dione (LKT Laboratories, Inc., St. Paul, MN, USA) was diffused in dimethyl sulfoxide (DMSO, suitable for hybridoma, Sigma Aldrich, Germany) to make 25 mM stock of the drug. Afterward, a decent amount of stock was compounded with a cell culture medium to achieve the composite’s appropriate concentration. The DMSO amount in the final solute used to perform incubation did not surpass 0.01% and it was affirmed that the peak amount did not statistically influence the cells.

### 4.3. Determination of Incorporation Efficiency and Characterization of Curcumin-Loaded Liposomes

Non-incorporated drug-crystals were separated from the curcumin-loaded liposomes during the liposome extrusion procedure (only curcumin-loaded liposomes can pass through Nucleopore polycarbonate filters). Additionally, the samples were centrifuged and then collected to ensure the absence of any free curcumin liposome samples. In total, 50 μL were taken before extrusion (initial) and after centrifugation. The lipid concentration was determined by the ammonium ferrothicyanate assay on a Varian Cary1 50 UV-Vis Spectrophotometer (Varian, Ltd., Victoria, Australia). The concentration of curcumin in the liposomes was determined photometrically at λ = 425 nm on the same spectrophotometer after the curcumin-loaded liposomes were dissolved in methanol. Curcumin encapsulation efficiency was 95 ± 1.6%. The size of the liposomes was 102 nm ± 2.3 and the polydispersity index was very low (0.051).

### 4.4. Curcumin-Mediated PDT Experimental Protocol

Cells were incubated with free or encapsulated curcumin (5, 10 μM) for 4 h according to Szlasa et al. [12] and Ambreen et al. [45] observations in FBS-free culture medium. Then the wells were washed twice with DPBS, fresh medium was added, and irradiation was performed using a halogen lamp (Penta Lamps, Teclas, Lugano, Switzerland) with the radiation power consistency set to 20 mW/cm^2^. The cells were irradiated for 2 min (2.5 J/cm^2^). The blue light (380−500 nm) was chosen to achieve the photodynamic effect (the light absorption peak of curcumin of 410 nm). Cells involved in curcumin and PDT treatment were protected from light at all times. After 24 h from irradiation, experiments were conducted according to the protocols.

### 4.5. Cell Viability Assay

The MTT assay is a colorimetric assay used to measure cellular metabolic activity to indicate cell viability, proliferation, and cytotoxicity. In the MTT assay, living cells transform yellow tetrazolium salt MTT into purple formazan crystals. This process is possible because living cells have an enzyme-mitochondrial dehydrogenase, which causes this change.

Cells were seeded at 8 × 10^4^ in 96-well culture plates and cultured as mentioned in the experiment description with curcumin and liposomal curcumin for 4 h in the dark. Different doses of curcumin and liposomal curcumin were experimentally established for the next experiments on MUG-Mel2, SCC-25, and HaCaT to obtain IC50. The MTT assay was performed after 24 h from irradiation. The MTT solution was added to the wells in a final concentration of 1 mg/mL for 3 h. Next, formazan dye was solubilized with 50 μL DMSO for 15 min. Absorbance was measured at 490 nm in BioTek Well-plate Reader (Winooski, VT, USA). The control group absorbance was 100%, whereas treated samples’ cell viability was counted using the formula: % = (A of experimental wells/A of the control wells) × 100%. After preliminary studies with different curcumin and liposomal-derivative doses (1, 2, 5, 10 μM) for the MTT assay, curcumin and liposomal curcumin was chosen in doses of 5 and 10 μM.

### 4.6. Wound-Healing Assay

A wound-healing assay was used to inquire cells’ interactions and cell migration. According to the manufacturer’s instructions, a wound-healing assay was made with the Culture-Insert 2 Well in μ-Dish 35 mm (Ibidi, Germany). The cells were seeded to achieve the monolayer in both parts of the insert. Following liposomal curcumin mediated PDT, the inserts were removed, the culture medium was exchanged, and the cells were cultured until about 100% confluency was reached in control cells. Control samples were without treatment at all. The photographs were taken after removal of the inserts at a time point 0 h and after 24 h of incubation by using a light microscope with a 10× magnifying objective (Olympus IX73 with a camera and CellSens Programme, Hamburg, Germany).

### 4.7. Flow Cytometry-Apoptosis Assay

Cells were drawn from each of the wells and transferred to Eppendorf tubes. Afterward, cells were centrifuged with PBS washing (7 min, 20 °C, 1000× *g*). The supernatant was gently removed and 1 mL of the Binding Buffer per 1 × 10^6^ cells was added. For the next step, 4 μL AAD-7 and 8 μL FITC was added to each sample, according to the manufacturer’s instruction. Eppendorf tubes were vortexed and incubated without the light for 15 min at room temperature. After incubation time, samples were analyzed with a flow cytometer using the FICT channel for Annexin 5 and PC5.5 channel for AAD-7 (Cytoflex, Beckman Coulter Life Sciences, Indianapolis, IN, USA). Negative samples were prepared without the staining and samples stained with one fluorochrome were used for compensation.

### 4.8. Immunocytochemistry (ICC) Staining for Apoptosis Detection

Cells were fixed with 4% paraformaldehyde for 10 min at room temperature, and then rinsed 2 × 5 min with PBS. Next, cells were blocked with endogenous peroxidase for 10 min using Peroxidase Blocking Reagent and rinsed with PBS 2 × 4 min. Non-specific proteins were blocked by Protein Block Serum-Free Ready to Use for 1 h. Following serum excess removal, anti-Bax and anti-Bcl-2 primary antibodies (Sigma-Aldrich) in dilution 1:200 were added on the slides for overnight incubation. Afterward, primary antibodies were rinsed with PBS for 2 × 4 min. A secondary rabbit antibody (Abcam, UK) in dilution 1:500 was added for 1 h at room temperature. After incubation time, cells were rinsed with PBS for 2 × 4 min and DAB Substrate in Chromogen Solution was added for 2–5 min until the light brown color was achieved. Cells were rinsed with distilled water for 2 × 4 min, and then hematoxylin was used for 1–2 min to stain cell nuclei. Next, cells were rinsed with tap water 2 × 5 min. The Fluoromount™ Aqueous Mounting Medium (Sigma Aldrich) was added onto the slides, and, the following day, the photographs were taken under the microscope (Olympus BX34 with camera DP74 and CellSens Programme, Hamburg, Germany). All ICC reagents were purchased from DAKO, Agilent (Glostrup, Denmark).

### 4.9. Statistical Analysis

All experiments were performed in triplicates and the values are presented as a mean ± standard deviation. Analysis between the groups was conducted using the non-parametric test Kruskal-Wallis for abnormal distributed data. a *p*-value below 0.05 was considered significant. PQStat Programme, version 1.8.2 (PQStat Software, Poland) was used for the calculations.

## 5. Conclusions

In conclusion, natural plant derivative-curcumin encapsulated in liposomes has been confirmed as a viable photosensitizer in PDT of skin cancer cell lines.

Improved bioavailability and increased stability revealed potent anti-cancer activity in squamous cell carcinoma and melanoma cell lines. The encapsulated compound preferentially accumulated in malignant skin cells. Contrarily, it showed decreased phototoxicity in normal skin keratinocytes HaCaT cells after PDT treatment. These results collectively support liposomal curcumin as a potential photosensitizer in developing natural-based photosensitizers that improve photodynamic therapy safety and efficacy. Thus, additional in vitro and in vivo studies on different normal and cancer cells are essential to confirm this less toxic natural plant derivative PS in the PDT approach.

## Data Availability

Data are contained within the article.

## Sample Availability

Samples of the compounds (curcumin) are available from the authors.
